# Development and Application of Wood Flour-Filled Polylactic Acid Composite Filament for 3D Printing

**DOI:** 10.3390/ma10040339

**Published:** 2017-03-24

**Authors:** Yubo Tao, Honglei Wang, Zelong Li, Peng Li, Sheldon Q. Shi

**Affiliations:** 1 College of Material Science and Engineering, Northeast Forestry University, Harbin 150040, China; tyblp@aliyun.com (Y.T.); whlmail@yahoo.com (H.W.); 2 Denton High School, Denton, TX 76203, USA; zelong.li.xdd@gmail.com; 3 Department of Mechanical and Energy Engineering, University of North Texas, Denton, TX 76203, USA; sheldon.shi@unt.edu

**Keywords:** wood flour, polylactic acid, composite filament, 3D printing, fused deposition modeling

## Abstract

This paper presents the development of wood flour (WF)-filled polylactic acid (PLA) composite filaments for a fused deposition modeling (FDM) process with the aim of application to 3D printing. The composite filament consists of wood flour (5 wt %) in a PLA matrix. The detailed formulation and characterization of the composite filament were investigated experimentally, including tensile properties, microstructure, thermogravimetric analysis (TGA), differential scanning calorimetry (DSC) and X-ray diffraction (XRD). The feedstock filaments of this composite were produced and used successfully in an assembled FDM 3D printer. The research concludes that compared with pure PLA filament, adding WF changed the microstructure of material fracture surface, the initial deformation resistance of the composite was enhanced, the starting thermal degradation temperature of the composite decreased slightly, and there were no effects on the melting temperature. The WF/PLA composite filament is suitable to be printed by the FDM process.

## 1. Introduction

Three-dimensional (3D) printing, also known as additive manufacturing (AM) technologies, creates three-dimensional objects in a layer-by-layer manner. Methods of 3D printing include fused deposition modeling (FDM), stereolithography apparatus (SLA), electron beam melting (EBM), laminated object manufacturing (LOM), selective laser sintering (SLS), digital light projection (DLP), etc. Over the past decade, AM technologies have extended to areas of the aerospace, automotive, medical, architecture, education, and fashion industries [1,2,3,4,5,6].

FDM is the most commonly used 3D printing methodology for its reliability, simplicity, affordability, minimal wastage and material availability. In the FDM process, thermoplastic filament, a conventionally used printable material, is fed to the liquefier head with the pressure generated from a driver gear and a grooved bearing. The 3D structures are printed layer by layer with the deposition of the filament material, which is heated to the melting temperature region and extruded through the extrusion nozzle. The liquefier head moves on the X-Y plane along software-generated tool paths and deposits the melted filament to form the printed part’s foundation on the print bed. After the completion of each layer, either the print bed or the extrusion nozzle will move and create space for the next layer’s fabrication. Every single layer will be stacked onto the previous layer until the part is completed [7,8].

Acrylonitrile butadiene styrene (ABS) and polylactic acid (PLA) filaments are widely used as feedstock for FDM. Polycarbonate (PC), polyamide (PA), and their mixtures are also viable materials [9,10]. Disadvantages of 3D structures built with FDM using pure plastic materials, such as high cost, low strength, and easy distortion, restrict the application of FDM in cost-effective, functional, load-bearing applications as well as in large-scale production [7]. Therefore, the development of new composite filaments for FDM is an important topic in the 3D printing industry.

Many researchers have performed studies to develop new composite filament materials for FDM such as iron powder/nylon [8], glass fibers/ABS [11], carbon nanotubes/ABS [12], carbon fibers/ABS [7], etc. However, there are few reported developments of composites reinforced or filled with plant fibers for expanding biomass materials to FDM 3D printing. Le Duigou et al. (2016) researched the effects of printing parameters on the mechanical properties using a named “Woodfill” filament supplied by the ColorFabb Company [13]. Girdis et al. (2017) presented microground macadamia nutshells and ABS plastics that were mixed to extrude filaments as feedstock of FDM 3D printers [14]. Filaments of PLA composites with bio-based materials are also on the market, such as “Laywoo (CC Products)” and “BambooFill (ColorFabb)”. Plant fiber/plastic matrix composites have special advantages including moderate density, easy degradation, and better economy. This new eco-material would also provide ideas for FDM applications in brand new areas.

Wood fiber/flour is one of the most popular raw materials for manufacturing plant fiber/plastic composites. PLA, a compostable synthetic polymer produced using monomer feedstock derived from corn starch, is an acceptable replacement for oil-derived plastics [15]. Integration of wood materials into the AM area is of interest due to its positive impacts on the environment and its better properties. Therefore, this paper presents the development of a wood flour (WF)-filled PLA composite filament for FDM 3D printing and its composite properties analysis.

## 2. Materials and Methods

### 2.1. Preparation of WF/PLA Composite Filament

The raw materials used in this paper were virgin PLA thermoplastic pellets (4032D, NatureWorks LLC, Minnetonka, MN, USA) and laboratory-made mean particle size 14 μm *Aspen* WF obtained from dried wood sawdust and grinded with a ball mill machine (QM0.4L, Chishun LLC, Nanjing, China). Taking into account that with the development of FDM, 3D printing will soon become low-cost and DIY (do it yourself), to a point that average users can even use self-made filaments. Therefore, a desktop-class plastic extruder (C2 model, Wellzoom LLC, Shenzhen, China) was used for fabricating pure PLA and WF/PLA composite filaments. This device consists of a single-screw extruder, with separate temperature (maximum 320 °C) controls for mixture and extrusion parts, which offers a maximum extrusion speed of 2 m·min^−1^.

The PLA pellets were initially dehydrated (103 °C) for four hours to eliminate moisture, then extruded to produce pure PLA filament. For the WF/PLA composite’s fabrication process, both the cut pure PLA filaments (size under 1 mm) and WF were dried (103 °C) to constant weight. WF and PLA particles were then blended with wood contents (5 wt %). During fabrication, 1.75 mm filaments were used, processing temperatures were set at 171 °C (extrusion part) and 175 °C (mixture part), with filament yield (extrusion) speed at 1 m·min^−1^.

Both pure PLA and WF/PLA composite filaments were printed as test specimens with a self-assembled FDM 3D printer (603S model, Shenzhen Aurora Technology Co., Ltd., Shenzhen, China). The nozzle diameter was 0.4 mm and nozzle temperature was set at 210 °C during print.

### 2.2. Properties Experiments of WF/PLA Composite Filament

Tensile properties of the specimens were measured by a universal testing machine (Changchun Kexin instruments Co., Changchun, China). The specimens were designed according to ASTM D638-03 [16]. Three replicated samples were prepared from each wood content for mean value calculation. Stress-strain curves and elasticity modulus were obtained.

A scanning electron microscope (SEM) (QUANTA200, FEI, Hillsboro, OR, USA) was used for examining the microstructure of gold coated filament specimens. The accelerating voltage was 5 kV. The SEM images were obtained at different zones on each sample.

TGA and DSC analysis of specimens were performed in a TA analyzer (Q 50, TA Instruments, New Castle, DE, USA) and DSC analyzer (Q 20, TA Instruments, USA). Samples were heated from 25 to 300 °C for TGA and to 400 °C for DSC with an increase rate of 10 °C·min^−1^ to observe their thermal degradation behaviors. Throughout the whole procedure, N_2_ flow rate was 30 mL·min^−1^.

Powder X-ray diffraction (XRD) measurements were recorded using D/max 220 analyzer (Rigaku, Tokyo, Japan). Samples were scanned under conditions of the Voltage 40 kV, current 30 mA, the starting angle of 5°, the termination angle of 40°, and step width of 0.02°.

## 3. Results and Discussion

### 3.1. Filament and 3D Product

**Figure 1:**
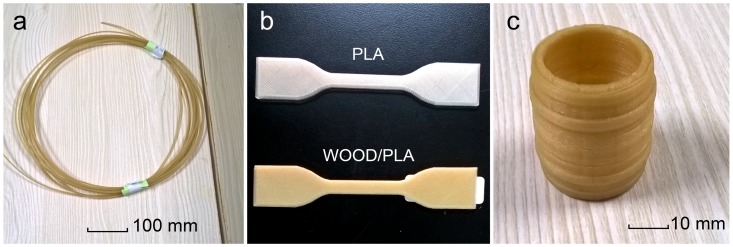
Filament, test specimens and 3D product: (**a**) WF/PLA composite filament; (**b**) specimens for tensile properties measurement; and (**c**) a barrel made by FDM 3D printer.

WF/PLA composite filaments were manufactured by the filament extruder with a diameter of 1.75 mm as shown in Figure 1a. Pure PLA filament can be obtained with the same method. The tensile test specimens of pure PLA and the WF/PLA composite are shown in Figure 1b. Adding WF (5 wt %) caused the composite to look more like a wooden material, especially when compared to pure PLA. A barrel was printed using the WF/PLA composite filament as shown in Figure 1c. Through experimentation and observation, the WF/PLA composite filaments were determined to be suitable for FDM printing.

### 3.2. Microstructure of Pure PLA and Composite Filament

**Figure 2:**
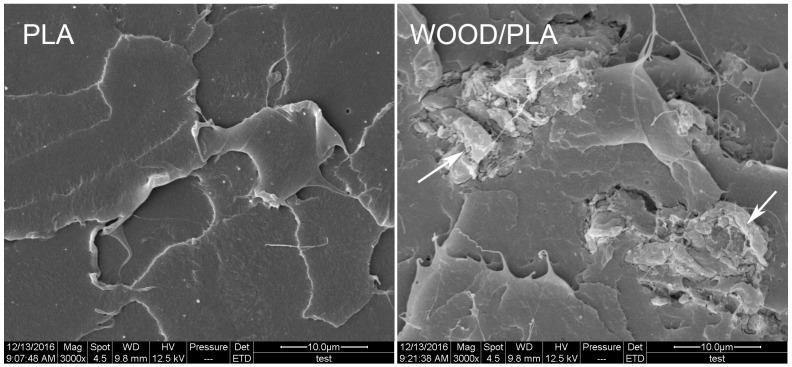
SEM of specimens (arrows indicate WF).

Figure 2 shows the microstructure of the specimens as well as the interfacial adhesion between the WF and PLA matrix. As shown in Figure 2, the fracture surface of pure PLA filament is flat and smooth. After adding WF, the composite fracture surface becomes rough. WF can be encapsulated by the PLA matrix. Clear gaps can be observed in certain areas between the WF and PLA interfaces. This indicates there is poor interfacial bonding between the PLA and WF. Due to the fact that WF is a polar surface (hydrophilic) and PLA is a non-polar surface (hydrophobic), in theory, the interface force between the WF and PLA is poor. Therefore, gaps occurred when the specimens were fractured. The interface force between the WF and PLA can be increased by improving the material compatibility, which is beneficial for improving the mechanical properties of the WF/PLA composite [17].

### 3.3. Properties of WF/PLA Composite Filament

**Figure 3:**
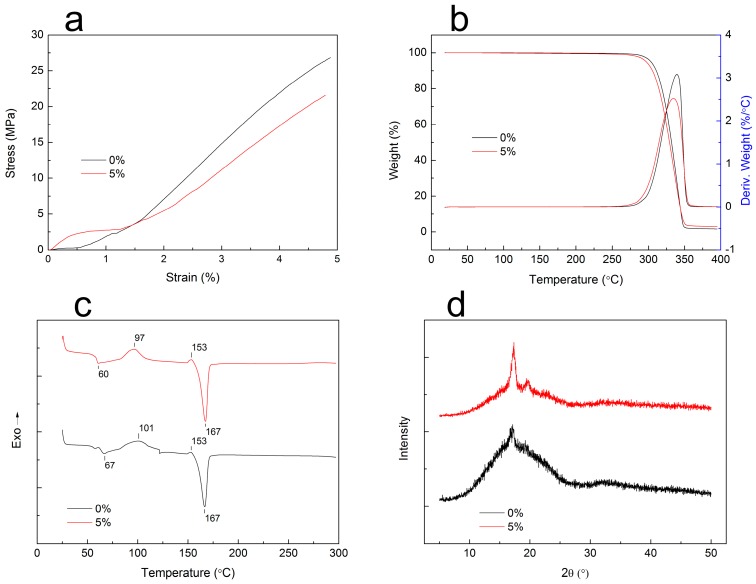
Specimen properties: (**a**) tensile strain-stress curves; (**b**) TGA-DTG curves; (**c**) DSC curves of specimens; and (**d**) XRD spectra.

Tensile properties: Tensile strain-stress curves of the specimens with different wood contents are illustrated in Figure 3a. In the strain range of 0%–1.5%, the tensile stress of specimens was increased by adding WF, indicating enhancement of the composites’ dimensional stability. However, when the strain percentage was above 1.5%, the strength of the WF/PLA composite was lower than pure PLA’s. The broken interface between the WF and PLA resulted in lowered composite stress under the same strain [18]. The data show that the elasticity moduli were increased by 30%, and similar results can be observed in the research of Petinakis et al. [17]. It is known that the tensile strength of a plastic specimen equals approximately 66% of its compressive strength. That is, the compressive strength of a specimen is higher than its tensile strength. For this reason, the WF/PLA composite filaments could be applied to structures requiring resistance against compressive force rather than tensile force.

TGA-DTG: The TGA curves in Figure 3b show that the wood flour has little effect on the thermal stability of the composites. With the addition of WF, the starting thermal degradation temperature of the composites decreased slightly and the final thermal decomposition residual ratio of the composites increased. The main reason for this phenomenon is that the thermal decomposition temperature of WF is lower and the thermal decomposition residual ratio is higher than that of PLA. Therefore, the composite’s starting decomposition temperature was decreased and the thermal decomposition residual ratio was increased [19]. At 270–350 °C, the composites decomposed swiftly. The DTG curve shows the maximum decomposition rate peaked around 330 °C, which was caused by the thermal degradation of the PLA molecular chain. After 350 °C, there was no more mass loss; this stage mostly represents the molecular chain carbonization process.

DSC: It can be seen from Figure 3c that the glass transition temperature and the cold crystallization temperature of PLA decreased with the addition of the WF. The glass transition temperature depends on the molecular characteristics, composition, and compatibility of the components in the composite [20]. Poor compatibility with added fillers can decrease the glass transition temperature [19]. Therefore, the decrease of the glass transition temperature, from 67 to 60 °C, can be ascribed to a weak interfacial adhesion between the WF and PLA due to poor compatibility. The presence of WF in the PLA matrix interfered with the crystallization process of the PLA and decreased the cold crystallization temperature from 101 °C to 97 °C. Lee et al. found that the crystallization enthalpy and the crystallinity of PLA can be decreased with the addition of WF. This may be partially caused by the inhibition effect of WF on the PLA crystal formation [14]. Figure 3c shows that the melting temperatures of the pure PLA filament and the WF/PLA composite filament are the same, at 167 °C.

XRD: Figure 3d depicts that the diffraction peak intensity of the composite increased significantly with the addition of the WF. This phenomenon indicates that the WF/PLA composite has more crystallinity than PLA [21]. This increased the interfacial tension between the WF and PLA and decreased the interfacial compatibility. In addition, the interaction between the WF and the PLA matrix restricted the movement of the molecular chains, leading to the formation of a rigid interface and decreasing the interface compatibility of the composite. The shoulder peak observed in the spectra shown in Figure 3d further illustrates that the interface compatibility is lower between the composite’s components.

## 4. Conclusions

WF/PLA composite filaments were produced and the properties measured in this study, and 3D specimens were printed using the FDM process. The following conclusions can be drawn from this study:

(1) The WF/PLA composite filament is suitable to be printed by the FDM process.
(2) Adding WF changed the microstructure of the PLA fracture surface, and the interfaces between the WF and PLA were obviously observable.
(3) The initial deformation resistance of the composite was enhanced after adding WF, compared to pure PLA.
(4) The starting thermal degradation temperature of the composites decreased slightly, and the final thermal decomposition residual ratio of the composites increased.
(5) Adding WF of 5 wt % has no effects on the melting temperature of the PLA.

In future work, the content ratio of wood and the interface compatibility between the WF and PLA need to be further improved.
